# Literary Notes

**Published:** 1898-01

**Authors:** 


### Literary Notes.

THE editors of the *Laryngoscope*, St. Louis, announce that, beginning with the January, 1898, issue, Messrs. John Wright & Co., of Bristol, Eng., will publish a foreign edition of that magazine. It is believed that this is a new departure in the field of American journalism and, we think, a distinct recognition of the advance made by the American medical profession in the trio of specialties represented as reflected through the pages of the journal in question.

THE *Georgia Journal of Medicine and Surgery*, edited by St. J. B. Graham, M. D., J. A. Van Marter, Jr., M. D., and W. E. Fitch, M. D., henceforth will contain from 75 to 100 pages instead of 50 pages as heretofore. The *Journal* has been improved in many ways, and the aim of the editors is to maintain the highest of professional standards. The publication office is 127–129 Liberty street west, Savannah, Ga., where all exchanges should be addressed.

THERAPEUTIC PROGRESS is the name of a monthly leaflet of eight pages published by Victor Koechl & Co., 79 Murray street, New York. It is devoted to a consideration of the newer remedies imported by this firm, among which may be mentioned argonin, benzosol, diphtheria antitoxine ("Behring"), tuberculin ("Koch") and others. Antipyrin, lanolin and dermatol are also included among the valuable preparations for which the above firm are sole agents for the United States. One particularly desirable feature of *Therapeutic Progress* is the condensation of reports and the like into as small a compass as possible, all useless elaboration and padding being eliminated. The publishers will be glad to mail copies regularly, free of charge, to any physician who may desire to have it. We have, from time to time, gleaned valuable and practical therapeutic hints from its perusal. Address Victor Koechl & Co., 79 Murray street, New York.

CURRENT THOUGHT is the name of a new quarterly published at Cleveland, O. Heretofore the publication has been issued as paper-covered books, called Current Thought Library. Mr. C. Elton Blanchard, the editor of this journal, was formerly publisher of the *Cleveland Medical Gazette* and is an active student of anthropological questions, being a lecturer and director of the American Institute of Anthropology. The medical profession will find much of interest in *Current Thought*, as will any thinking man or woman. Specimen copies will be sent upon request.

---

The *Albany Medical Annals* for February, 1898, will be an extra number upon subjects pertaining to hospitals—their construction and administration. The following articles will be included in this publication : A description of hospital buildings on the pavilion plan, by Albert Vander Veer, M. D., attending surgeon Albany Hospital ; The construction of hospitals, by P. M. Wise, M. D., president New York State Commission in Lunacy ; The warming and ventilation of hospitals, by Fred. P. Smith, heating engineer for New York State Architect and Capitol Commissioners, Albany, N. Y.; The plumbing of hospitals, by Wm. Paul Gerhard, C. E., consulting engineer for sanitary works, New York ; Hospital equipment, by C. Irving Fisher, M. D., superintendent of the Presbyterian Hospital, New York ; The medical service of hospitals, by Henry M. Hurd, M. D., medical superintendent of the Johns Hopkins Hospital, Baltimore. These papers will be appropriately illustrated. The reputations of the contributors are so well known that the latest accepted ideas upon the subjects treated are assured and a variety of practical topics, not elsewhere to be found, will be presented for reference to all who are interested in the management of hospitals. The price of this number of the *Annals* to nonsubscribers will be 25 cents, post-paid. All orders should be addressed *Albany Medical Annals*, Albany, N. Y.

---

The *International Medical Magazine*, edited by Dr. Walter L. Pyle, has removed its office from 3709 Spruce street to Professional Building, 1831–33 Chestnut street, Philadelphia. Exchanges are requested to make a note of this on their mail lists.

---

M. J. Breitenbach Company, 56–58 Warren street, New York, have our thanks for a valuable year-book containing separate pages for daily memoranda during the year 1898, suitable for the physician's desk. It is one of the most convenient books of its kind we have seen.

